# Physical Distancing and Social Connectedness Among Older Adults

**DOI:** 10.1093/geroni/igab046.136

**Published:** 2021-12-17

**Authors:** Julie Lutz, Emily Bower, Ellen Beckwith, Julie Choi, Kim Van Orden

**Affiliations:** 1 VA Palo Alto Health Care System, Palo Alto, California, United States; 2 Pacific University, Hillsboro, Oregon, United States; 3 University of Rochester Medical Center, Rochester, New York, United States; 4 University of Rochester School of Medicine & Dentistry, Rochester, New York, United States

## Abstract

The COVID-19 pandemic has significantly impacted older adults; due to elevated risk, many older adults have followed physical distancing guidelines. These efforts, while critical to public health, have also impacted the social interactions and connectedness of older adults. In this mixed-methods study, we conducted qualitative interviews and administered questionnaires to 23 adults age 60 and older to examine how physical distancing has affected their social connectedness; what strategies and supports they have utilized to maintain or improve social connectedness despite physical distancing; and what types of supports, programs, and interventions they feel could promote and foster social connectedness among older adults during physical distancing. The results may have implications not only for the pandemic, but also for older adults who cannot leave their homes or experience barriers to typical social activities for any reason (e.g., being homebound, having functional impairments).

